# Combined rectus sheath block with transverse abdominis plane block by one puncture for analgesia after laparoscopic upper abdominal surgery: a randomized controlled prospective study

**DOI:** 10.1186/s12871-024-02444-6

**Published:** 2024-02-09

**Authors:** Shan Yu, Yaling Wen, Jing Lin, Jinghao Yang, Yihang He, Youbo Zuo

**Affiliations:** 1 Department of Anesthesiology, Affiliated Hospital of North Sichuan Medical College, Nanchong, 637000 China; 2 Department of Anesthesiology, Armed Police Forces Hospital of Sichuan, Leshan, 614000 China

**Keywords:** Ultrasound, Upper abdominal surgery, Analgesia, Rectus sheath block, Transversus Abdominis plane block

## Abstract

**Background:**

Rectus sheath block (RSB) and transversus abdominis plane block (TAPB) have been shown to reduce opioid consumption and decrease postoperative pain scores in abdominal surgeries. However, there are no reports about the one-puncture technique of RSB combined with TAPB for perioperative pain management during laparoscopic upper abdominal surgery.

**Methods:**

A total of 58 patients were randomly assigned to the control group (C), the TAP group (T), and the one-puncture technique of RSB combined with TAPB group (RT). The patients in group C did not receive any regional block. The patients in group T received ultrasound-guided subcostal TAPB with 30 mL of 0.33% ropivacaine on each side. The patients in the RT group received a combination of RSB and TAPB with 15 mL of 0.33% ropivacaine in each plane by one puncture technique. All patients received postoperative patient-controlled intravenous analgesia (PCIA) after surgeries. The range of blocks was recorded 20 min after the completion of the regional block. The postoperative opioid consumption, pain scores, and recovery data were recorded, including the incidence of emergence agitation (EA), the times of first exhaust and off-bed activity, the incidence of postoperative nausea and vomiting, dizziness.

**Results:**

The range of the one-puncture technique in group RT covered all areas of surgical incisions. The visual analogue scale (VAS) score of the RT group is significantly lower at rest and during coughing compared to groups T and C at 4, 8, 12, and 24 h after surgery, respectively (*P* < 0.05). The consumption of sufentanil and the number of postoperative compressions of the analgesic pumps at 24 and 48 h in the RT group are significantly lower than those in groups T and C (*P* < 0.05). The incidence of EA in the RT group is significantly lower than that in groups T and C (*P* < 0.05).

**Conclusion:**

The one-puncture technique of RSB combined with TAPB provides effective postoperative analgesia for laparoscopic upper abdominal surgery, reduces the incidence of EA during PACU, and promotes early recovery.

**Trial registration:**

ChiCTR, ChiCTR2300067271. Registered 3 Jan 2023, http://www.chictr.org.cn.

**Supplementary Information:**

The online version contains supplementary material available at 10.1186/s12871-024-02444-6.

## Background

Laparoscopic surgery has emerged as the preferred technique for upper abdominal surgeries in order to minimize trauma, as it requires only a minimal skin incision. However, the incisions for laparoscopic surgery are widely distributed. Postoperative pain remains the most significant factor influencing patients’ recovery. Inadequate analgesia may increase the risk of postoperative complications, such as atelectasis, organ dysfunction, and chronic pain [1–2]. Opioid analgesia is commonly used following laparoscopic abdominal surgery [3]. However, high doses of opioids can lead to postoperative nausea and vomiting, affect intestinal function recovery, prolong hospital stays, and decrease patient satisfaction [4].

There has been an increasing use of ultrasound (US) guidance for peripheral nerve blocks to provide postoperative multimodal analgesia. US-guided rectus sheath block (RSB) and transversus abdominis plane block (TAPB) have been used in abdominal surgeries, demonstrating their potent analgesic effects [5–6]. Previous studies have shown that US-guided TAPB combined with RSB produced better analgesic effects compared to TAPB or RSB alone in abdominal surgery [7–8]. Currently, multipoint nerve block methods are typically utilized in clinical practice, but they are complex and can exacerbate the patient’s pain. However, there is no evidence in the literature to support the use of the US-guided one-puncture technique of RSB combined with TAPB. Based on the location of the incision in laparoscopic upper abdominal surgery and the neuromuscular anatomy, a novel approach is presented: US-guided one-puncture of rectus sheath block combined with transverse abdominis plane block.

The aim of this study is to evaluate the efficacy of postoperative pain management by US-guided one-puncture technique of RSB combined with TAPB in patients following laparoscopic upper abdominal surgery.

## Methods

### Patients

This randomized, single-center trial study was approved by the Research Ethics Committee of the Affiliated Hospital of North Sichuan Medical College (approval no. 2022ER508-1) and written informed consent was obtained from all subjects participating in the trial. The trial was registered with the Chinese registry of clinical trials at http://www.chictr.org.cn (Registration number: ChiCTR2300067271, date of registration: 03/01/2023) before patient enrollment. This study adhered to the applicable Consolidated Standards of Reporting Trials (CONSORT) guidelines and was performed from 8 Jan 2023 and 12 May 2023.

We recruited patients between 18 and 79 year old who were undergoing elective laparoscopic upper abdominal surgery at the Affiliated Hospital of North Sichuan Medical College. To be enrolled, patients had to have an American Society of Anesthesiologists (ASA) physical status of I to III and a body mass index (BMI) ranging from 18 to 30 kg/m2. Patients were excluded if they had a poor understanding of the research process and assessment, allergies to drugs relevant to the study, a history of local skin infection in the operator area of the nerve block, a history of significant pain or painful disease, mental illness, alcoholism, or long-term use of analgesic medication (continuous use for more than 3 months). The exit criteria were that the laparoscopic surgery could not be completed and had to be converted to laparotomy, and patients who were transferred to the ICU after surgery.

### Randomization and blinding

Patients were randomly assigned to the control group (C), the TAP group (T), and the one-puncture technique of RSB combined with TAPB group (RT). The patients in group C did not receive any regional block. The patients in group T received ultrasound-guided bilateral subcostal TAPB with 30 mL of 0.33% ropivacaine (Ruiyang, Shandong, China). The patients in the RT group received a combination of ultrasound-guided RSB and TAPB with 15 mL of 0.33% ropivacaine in each plane by one puncture technique. Patients in all groups received postoperative patient-controlled intravenous analgesia (PCIA) after surgery.

The randomization was conducted using randomization numbers generated by SPSS. The randomized results were placed in a sealed envelope and sent to an independent anesthesiologist, who then prepared the drug on the morning of the operation. The staff responsible for data collection and analysis were unaware of the group allocations.

### General anesthesia and monitoring

After the patient entered the operating room, peripheral venous access was routinely established. Pulse oximetry, electrocardiogram, and non-invasive blood pressure were monitored, and the radial artery cannula was inserted under local anesthesia to monitor invasive arterial pressure. Patients in the RT and T groups received US-guided bilateral RSB and TAPB under sedation and analgesia before anesthesia induction (midazolam 1 mg and sufentanil 5 µg), while group C did not receive any regional block. Anesthesia induction was carried out after testing the blocking range with IV midazolam (Enhua, Jiangsu, China) 0.03 mg/kg, sufentanil (Enhua, Jiangsu, China) 0.3–0.5 µg/kg, etomidate (Enhua, Jiangsu, China) 0.2–0.3 mg/kg, and cisatracurium (Jianyou, Nanjing, China) 0.15 mg/kg. Tracheal intubation was performed 3–5 min after induction. Anesthesia was maintained using inhalational sevoflurane (Hengrui, Shanghai, China) with a target of age-adjusted minimum alveolar concentration (MAC) of 1.0–1.5 in an air and oxygen mixture with an inspired oxygen fraction (FiO2) of 50%. Intermittent intravenous injections of sufentanil and cisatracurium (0.05 mg/kg every 30 min) were also administered. The lungs were ventilated with a tidal volume of 8 mL/kg, and the breathing frequency was adjusted to maintain an end-tidal carbon dioxide partial pressure of 30 to 40 mmHg. The anesthetic dosage was continuously adjusted based on the Bispectral Index (BIS) value, which was maintained between 40 and 60, and the mean arterial pressure, which was kept within 20% of the baseline value. Ephedrine (Shuanghe, Beijing, China) was administered if the blood pressure dropped by more than 20% below the baseline, and atropine (Changjiang, Anhui, China) was administered if the heart rate fell below 50 bpm.

### Postoperative care

The PCIA was initiated in the post-anesthesia care unit (PACU). The PCIA regimen comprised 3 µg/kg of sufentanil, 10 mg of tropisetron, and normal saline to a total volume of 150 mL, with a background infusion rate of 2 mL/h, a bolus dose of 2 mL, a lockout interval of 15 min, and a maximum limit of 10 mL within 1 h. Intramuscular tramadol 100 mg was administered as an additional rescue analgesic.

### Laparoscopic procedures

**Fig. 1:**
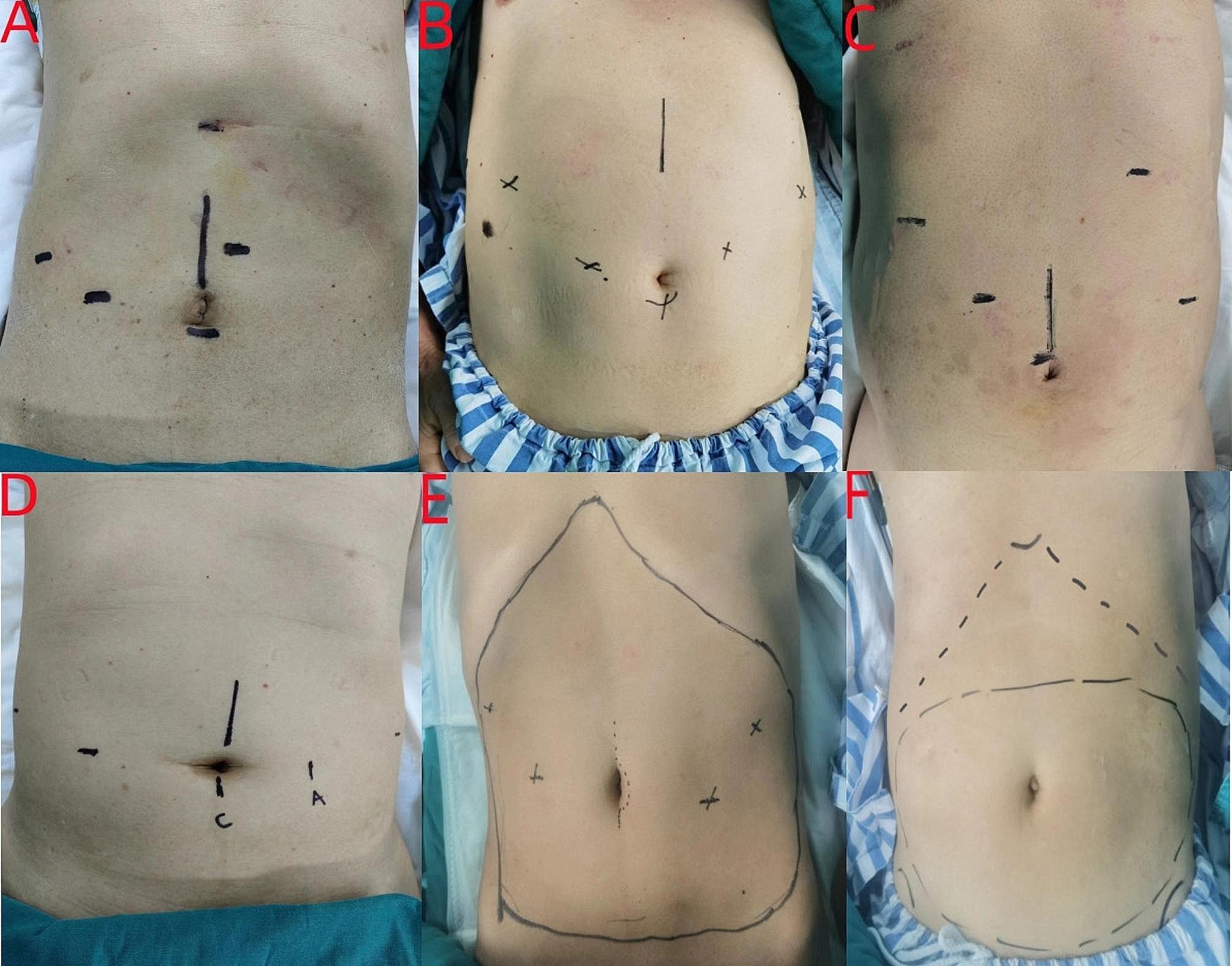
Laparoscopic incision and trocar insertion using the five-hole method and the block plane of the one-puncture RSB + TAPB or the subcostal TAPB. **A**–**D** are the positions of the holes and the specimen removal for laparoscopic hepatic lobectomy, radical gastrectomy, splenectomy, and pancreatic surgery respectively. **E** is the block plane range of the one-puncture RSB + TAPB. **F** is the block plane range of the subcostal TAPB (Costal margin is indicated by the black dotted line)

The study included laparoscopic hepatic lobectomy, radical gastrectomy, splenectomy, and pancreatic surgery. The pneumoperitoneum was created using carbon dioxide at a pressure ranging from 12 to 15 mmHg. According to the laparoscopic surgical guidelines, the five-port method was commonly used (Fig. 1). The puncture incision was located in the mid-upper ventral region, extending from the bilateral costal margin and xiphoid to the umbilicus. The incision was typically made either under the xiphoid process or around the umbilicus for specimen removal.

### US-guided RSB and TAPB

**Fig. 2:**
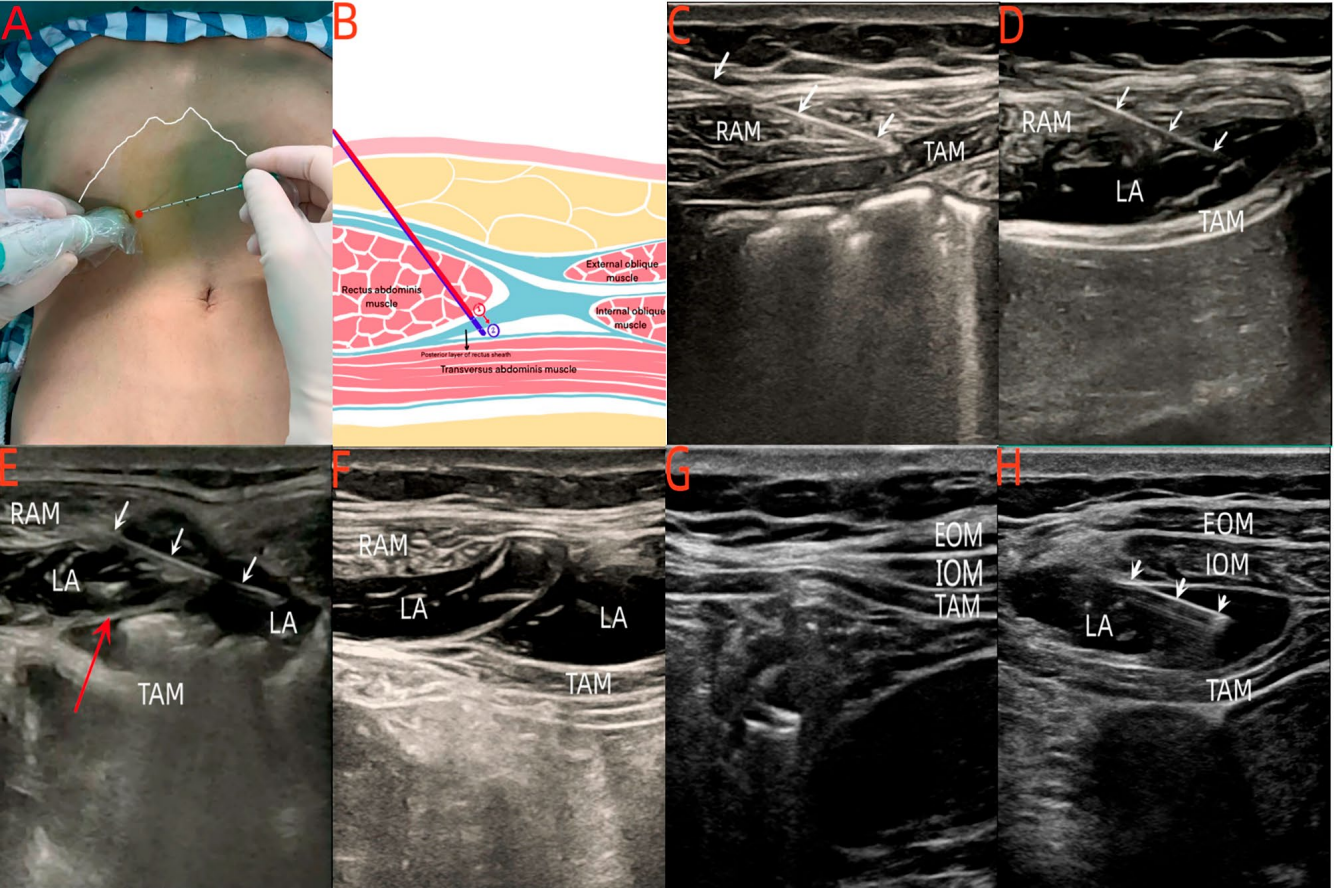
Procedures for one puncture of the RSB + TAPB block and the subcostal TAP block. **A**: Graphic representing probe position in the undercostal about one-puncture of RSB and TAPB (The red point is the needle point; Costal margin is indicated by the white line). **B**: Anatomical diagram of one-puncture of RSB and TAPB (The red line is the block path of RSB; The blue line is the block path of TAPB). **C**: The ultrasound images of one-puncture of RSB and TAPB injection path (White arrows indicate the needle injection path). **D**: The ultrasound images of the RSB by the one-puncture nerve block. **E**: The ultrasound images of continuing to break through the posterior layer of the rectus abdominis sheath by the one-puncture nerve block (The red arrow indicates the posterior layer of the rectus abdominis sheath). **F**: The ultrasound images of the spread of local anesthetic after a successful one-puncture nerve block. **G**: The ultrasound images of the subcostal TAP. **H**: The ultrasound images of the spread of local anesthetic after a successful subcostal TAP nerve block. RAM, the rectus abdominal muscle. EOM, the external oblique muscle. IOM, the internal oblique muscle. TAM, the transverse abdominis muscle. LA, local anesthetic

The patients in the RT group received a one-puncture technique of RSB combined with TAPB. The procedure was carried out by a certified anesthesiologist (Jing Ling) using US guidance (Mindray M9 Ultrasound System) and a high-frequency linear ultrasound probe. In all instances, a second investigator (Yaling Wen) was responsible for observing the images and confirming the blocking process. The probe was positioned transversely in the midline of the abdomen, between the xiphoid process and the umbilicus, revealing the linea alba. It was then moved outward along the costal margin, demonstrating the rectus abdominis overlapping the transverse abdominis (Fig. 2B and C). A 22-gauge, 120-mm ultrasound-visible block needle (Stimuplex® B-Braun medical, Melsungen, Germany) was inserted from the inner side (Fig. 2A). Under direct vision, we reached the posterior rectus abdominis sheath and pierced the anterior layer of the posterior sheath. Saline was injected to adjust its position. After that, 15 mL of 0.33% ropivacaine was administered (aspiration was performed for every 5 mL injection), and we observed the local anesthetic spreading inward (Fig. 2D). Then, the needle broke through the posterior layer of the tendon, and saline was injected to confirm the needle’s placement in the transversus abdominis plane. After confirming the tip placement, 15 mL of 0.33% ropivacaine was slowly injected. The needle tip was advanced along the plane, which was expanded by the local anesthetic to block a wider area (Fig. 2E). The signs of local anesthetic toxicity, such as tinnitus, numbness around the mouth, and slurred speech were strictly monitored. After completing the block, a double membrane capsule with ultrasound signs would be found (Fig. 2F). The contralateral nerve block was performed using the same method and volume of anesthetic solution (Additional file 1).

The patients in group T received only US-guided TAPB. For the TAPB, the probe was moved outward along the costal margin until the external oblique muscle, internal oblique muscle, and transverse abdominis muscle were visible (Fig. 2G) [9]. The needle was inserted in-plane until the tip was positioned in the plane between the internal oblique muscle and the transverse abdominis muscle. Then, 30 mL of 0.33% ropivacaine was injected (Fig. 2H). The contralateral nerve block was performed using the same method and volume of anesthetic solution.

### Data collection

Sensory assessment was conducted using a 75% ethyl alcohol swab and a pinprick test on the abdominal wall 20 min after the regional block was completed. Effective analgesic efficacy was defined as the loss of cold temperature sensation to an alcohol swab or the loss of pinprick pain sensation compared with the non-blocked area. The loss of sensation was assessed by examining the anatomic distribution of intercostal nerves from the sixth thoracic spine to the first lumbar spine, and recording the level at the anterior median line, midclavicular line, and anterior axillary line. It was recorded by a blinded member of the research team. Patients who experienced a failed block were excluded from the study. Sufentanil-based PCIA was administered to all patients using the same regimen for 48 h after surgery. The primary outcome was the cumulative sufentanil usage during two time periods after the operation (0 to 24 h, 0 to 48 h), and it was recorded by another blinded member of the research team. The secondary outcomes included postoperative pain intensity at rest and during coughing on the visual analogue scale (VAS) at 4, 6, 8, 12, 24, and 48 h post-operation, with 0 indicating no pain and 10 indicating the worst possible pain.

The incidence of emergence agitation (EA) was evaluated in the post-anesthesia care unit (PACU) using the Richmond Agitation Sedation Scale (RASS). Patients with a RASS score ≥ 1 were classified as experiencing EA [10]. The administration of intraoperative medications and postoperative rescue analgesics, the frequency of postoperative use of the analgesic pump, time to first exhaust and off-bed activity, as well as the occurrence of postoperative nausea and vomiting (PONV) and dizziness were also documented.

### Statistical analysis

The sample size was determined based on the pre-experimental data collected before the study. The means ± standard deviations of sufentanil used during the first 24 h period after surgery were recorded as follows: Group C: 57.51 ± 10.66 µg, Group RT: 45.82 ± 12.99 µg. Based on a significance level of 5%, a power of 80%, and a 20% dropout rate, a minimum of 20 patients per group were enrolled.

Statistical analysis was conducted using SPSS 26.0 (SPSS Inc., Chicago, IL). The normality of the measurement data was analyzed using the Shapiro-Wilk test. Normally distributed data are typically presented as means ± standard deviations and were analyzed using an ANOVA test. Non-normally distributed data are presented as medians and interquartile ranges, and the Kruskal-Wallis test was used to compare the differences between and within the groups. Categorical data were reported as numbers and percentages and analyzed using the χ2 test or Fisher’s exact test. *P* values less than 0.05 were deemed significant.

## Results

**Fig. 3:**
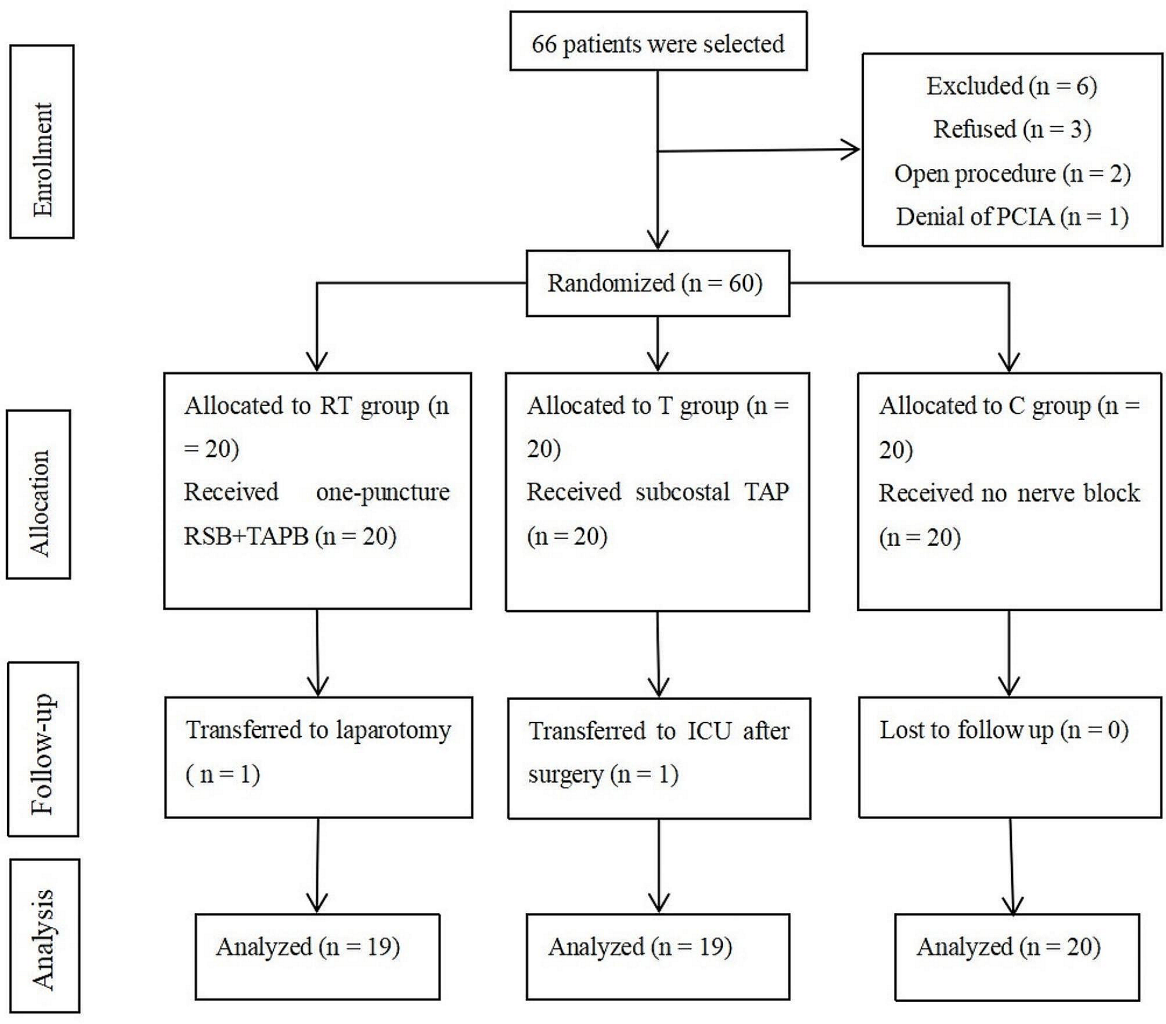
Flow diagram of all patients

A total of 66 patients were included in this study. Six patients were excluded. The remaining 60 patients were randomly assigned to groups C, T, and RT (*n* = 20). During the trial, one patient in the RT group underwent laparotomy, while the other patient in the T group was transferred to the ICU after surgery. We analyzed the remaining 58 eligible patients (Fig. 3). The three groups showed no significant differences in demographic parameters, surgical conditions, fluid and anesthetic administration (*P* > 0.05, Table 1).

**Table 1:** The demographic data and perioperative characteristics of the three groups

Variables	Group C (*n* = 20)	Group T (*n* = 19)	Group RT (*n* = 19)	*P* value
Age, yrs, median (IQR)	62.5 (59, 70)	65 (50, 68)	59 (44, 68)	0.80
Sex, n (%)				0.15
Male	10	13	15	
Female	10	6	4	
BMI, kg/m^2^, mean (SD)	22.1 (2.3)	23.6 (2.4)	22.8 (1.9)	0.11
ASA class, n (%)				0.57
II	10	7	10	
III	10	12	9	
Comorbidities, n (%)				0.93
hypertension	6	5	6	
Type of surgery, n (%)				1
Hepatic lobectomy	11 (55.0)	10 (52.6)	10 (52.6)	
Radical gastrectomy	6 (30.0)	6 (31.6)	6 (31.6)	
Splenectomy	1 (5.0)	1 (5.3)	0 (0.0)	
Pancreatic surgery	2 (10.0)	2 (10.5)	3 (15.8)	
Anesthesia time, min, mean (SD)	283.5 (84.62)	278.53 (100.31)	297.63 (82.54)	0.79
Operation time, min, median (IQR)	215 (176.3, 241.3)	160 (140, 235)	215 (170, 255)	0.31
Sufentanil, µg, median (IQR)	60 (50, 70)	60 (50, 60)	55 (50, 60)	0.43

Variables are expressed as Mean (SD), number or Median (IQR). BMI body mass index, ASA American Society of Anesthesiology, SD standard deviations, IQR interquartile range

The dermatomal distribution of skin sensory loss was recorded 20 min after the block in groups T and RT (Fig. 1). The block plane (the block range at each location was satisfied by more than 50% of patients) of the RT group is concentrated in the anterior median line, midclavicular line, and anterior axillary line at T6–T11, T7–T11, and T8–T11. The block plane of the T group is concentrated in the anterior median line, midclavicular line, and anterior axillary line at T8–T12, T8–T12, and T9–T11. As can be seen from the location of the laparoscopic hole and specimen removal for the four types of surgeries in this study, the block plane in the RT group covers all the incision positions, while the block range of the T group is less than the RT group, particularly below the xiphoid (Fig. 1E and F).

**Fig. 4:**
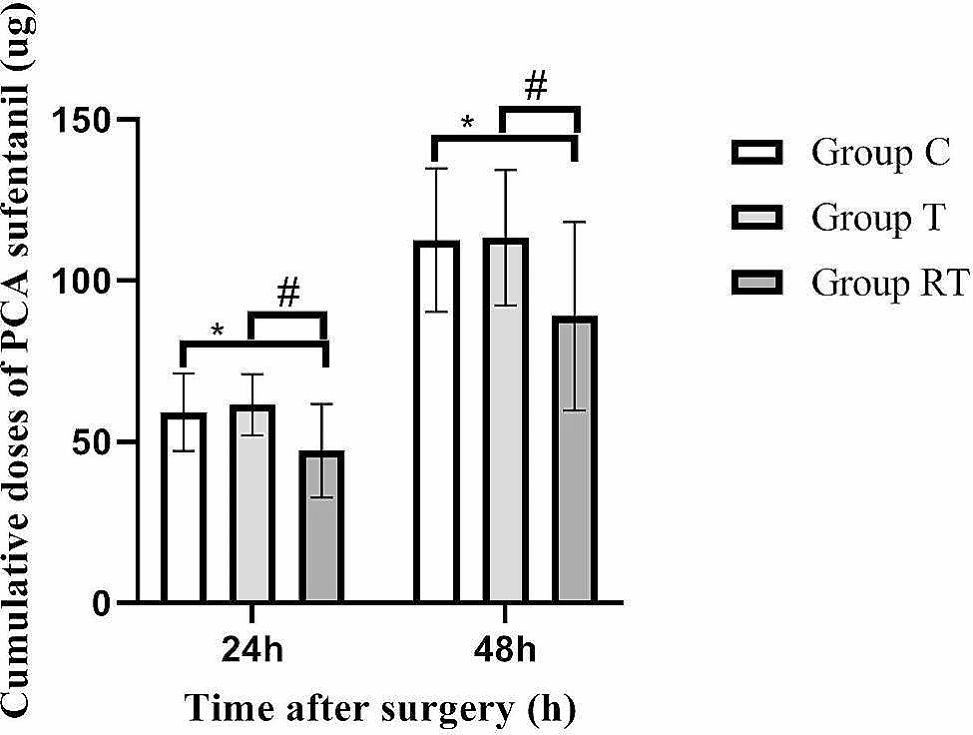
Postoperative sufentanil use in the three groups. *p < 0.05 vs. Group C, ^#^*p* < 0.05 vs. Group T

**Fig. 5:**
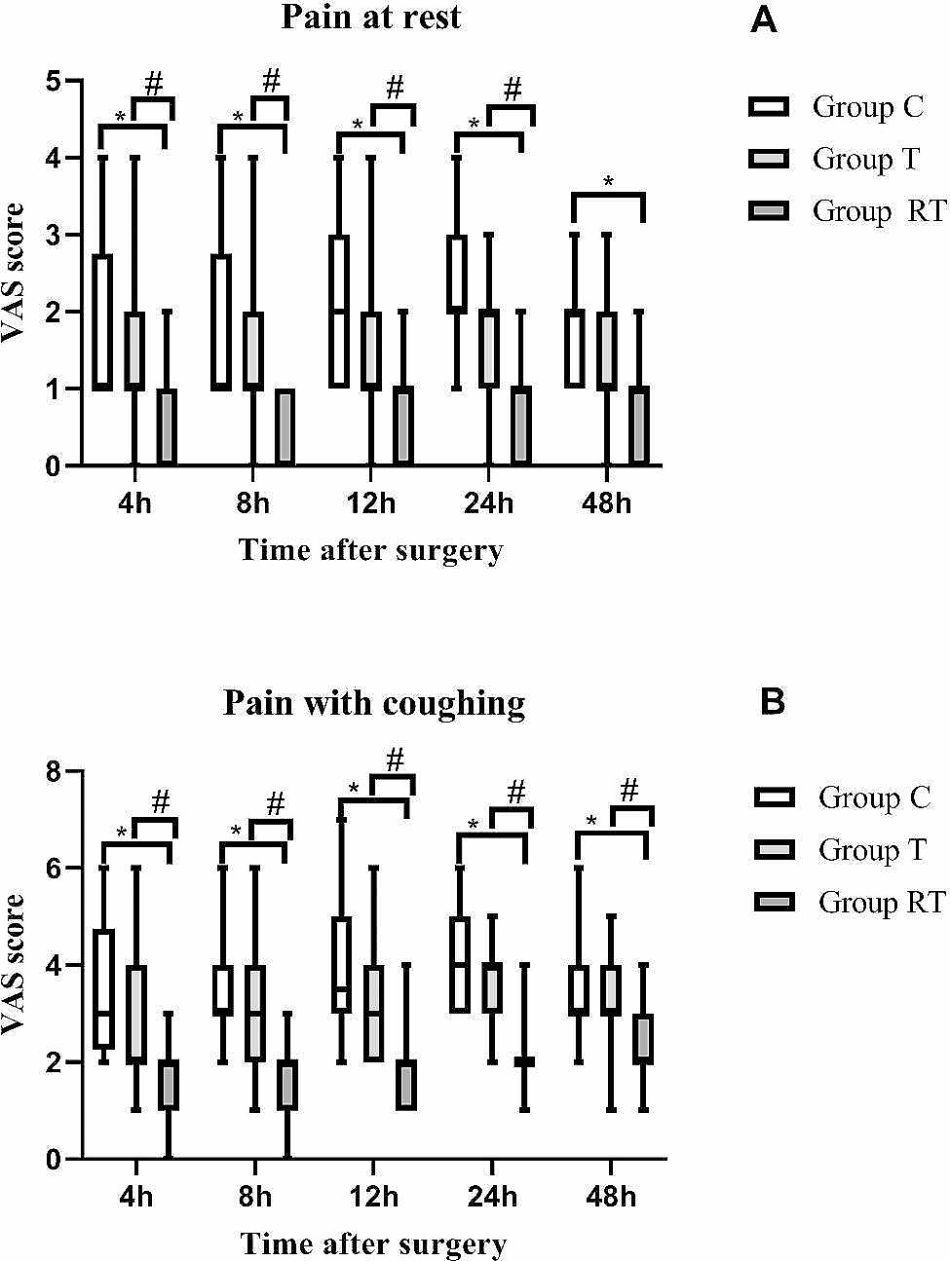
**A**: Postoperative pain scores at rest in the three groups (VAS = visual analogue scale). **B**: Postoperative pain scores with coughing in the three groups. *p < 0.05 vs. Group C, ^#^*p* < 0.05 vs. Group T

The RT group consumed significantly less postoperative sufentanil than the C and T groups at 0 to 24 h (47.20 ± 14.46 µg vs. 61.39 ± 9.49 µg vs. 59.11 ± 12.09 µg, *P* < 0.05), 0 to 48 h (88.89 ± 29.25 µg vs. 113.29 ± 20.99 µg vs. 112.56 ± 22.28 µg, *P* < 0.05). The C and T groups showed no significant difference in the usage of postoperative sufentanil (*P* > 0.05, Fig. 4). The postoperative VAS pain scores at rest and during coughing in the RT group were significantly lower than those in groups T and C at 4 h, 8 h, 12 h, and 24 h, respectively (*P* < 0.05, Fig. 5). The T and C groups did not show statistically significant differences (*P* > 0.05).

In PACU, the incidence of EA in the group RT were statistically lower than that in groups T and C (*P* < 0.05, Table 2). The number of postoperative compressions of the analgesic pumps was significantly less in the RT group than those in groups T and C during 24 and 48 h, and the T and C groups were not statistically significant differences (*P* < 0.05, Table 2). Although the number of patients reporting PONV during the 48 h following surgery was lower in the RT group, the differences among groups were not statistically significant. The incidence of dizziness in the RT and T groups was significantly lower than that in group C (*P* < 0.05, Table 2). Compared to group C, the time to first exhaust and first off-bed activity were shorter in groups T and RT, but the differences between groups T and RT were not statistically significant (*P* < 0.05, Table 2).

**Table 2:** Comparisons of postoperative recovery of patients in the 3 groups

Variables	Group C (*n* = 20)	Group T (*n* = 19)	Group RT (*n* = 19)	*P* value
the incidence of EA in PACU, n (%)	16 (55.2%)	11 (37.9%)	2 (6.9%) ^*#^	< 0.001
the number of compressions at 24 h, median (IQR)	2 (1, 6.25)	2 (0, 3)	0 (0, 1)^*#^	0.001
the number of compressions at 48 h, median (IQR)	3.5 (1.25, 7)	3 (2, 4)	0 (0, 1)^*#^	< 0.001
Rescue analgesic, n (%)	2 (10%)	4 (21.1%)	1 (5.3%)	0.37
PONV, n (%)	8 (40%)	8 (40%)	3 (15.8%)	0.17
Dizziness, n (%)	8 (40%)	2 (10.5%)^*^	0 (0%)^*^	0.002
Time to first exhaust, h, mean (SD)	72.35 (18.26)	53.6 (18.51)^*^	52.79 (16.91)^*^	0.001
Time to off-bed activity, h, mean (SD)	62.23 (17.89)	41.63 (20.83)^*^	38.32 (16.72)^*^	< 0.001

Variables are expressed as Mean (SD), number or Median (IQR). PACU Post-anesthesia care unit,

PONV Postoperative nausea and vomiting, SD standard deviations, IQR interquartile range

*P < 0.05 versus Group C; #*P* < 0.05 versus Group T

## Discussion

In the prospective study, a one-puncture technique of RSB combined with TAPB was proposed and administered. We found that the US-guided one-puncture technique of RSB combined with TAPB could meet the analgesic requirements of most upper abdominal surgeries. This approach significantly reduced analgesic consumption after surgery, ameliorated postoperative pain intensity of laparoscopic upper abdominal surgeries, and promoted rapid recovery in patients after surgery.

As the core of enhanced recovery after surgery, laparoscopic surgery was launched to minimize the traumatic effects of incisions and has been increasingly popular for upper abdominal surgery [11–12]. However, postoperative pain is still the primary factor affecting patients’ recovery after laparoscopic upper abdominal surgery [13], and adequate postoperative analgesia remains essential [14]. Multimodal analgesia, especially US-guided peripheral nerve block, has proven safety and effectiveness for pain management [15–16].

TAPB can theoretically block the T7–L1 nerves [17]. Wu et al. concluded that TAPB in laparoscopic colorectal surgery reduced the rest pain score at 6 h after surgery and the use of analgesics at 24 h after surgery [18]. Among these techniques, the subcostal TAP is more suitable for upper abdominal surgery [19], Ma et al. reported that subcostal TAPB with a 0.25% levobupivacaine dose of 0.5mL/kg provided effective analgesia in the anterior abdominal wall from the medioventral line to the anterior axillary line [20]. Additionally, Yoon et al. illustrated that ultrasound-guided bilateral subcostal TAPB provides effective postoperative analgesia and reduces opioid consumption after laparoscopic gastrectomy [21]. However, in this experiment, the subcostal TAPB did not significantly reduce the postoperative analgesic drug usage or the postoperative analgesic scores for laparoscopic upper abdominal surgery. The conclusions are consistent with previous studies [10, 22].

It was found that US-guided bilateral TAPB combined with RSB provided effective postoperative analgesia for upper abdominal surgery and reduced postoperative opioid use, shortened postoperative hospital stay without increasing the incidence of opioid-related adverse events [23–26]. However, for two-sided nerve blocks, this combination technique typically requires four instances of ultrasound localization and four puncture points. It would prolong the duration of the blocking operation, increasing the discomfort and tension of conscious patients. Due to the special nature of the tips of certain nerve block needles, it can also increase the difficulty of inserting through the skin when puncturing multiple times. Based on the anatomy, under the costal margin, the transverse abdominis is located both below the rectus abdominis muscle and the abdominal internal oblique muscle, the rectus abdominis muscle is adjacent to the abdominal internal oblique muscle (Fig. 2B). Theoretically, injecting drugs completes the rectus sheath block between the rectus abdominis muscle and its posterior sheath. By breaching the tendon’s posterior layer, the transverse abdominis plane block can be accomplished. Therefore, we propose the one-puncture technique for RSB combined with TAPB, with the aim of potentially replacing the conventional combination blockade.

This study investigates the range of the block plane in the one-puncture method, which covers all positions of the puncture holes and the location of specimen removal during laparoscopic upper abdominal surgery. The block plane of one-puncture technique was better than the subcostal TAPB, especially when positioned under the xiphoid. Additionally, the one-puncture technique of RSB combined with TAPB resulted in significantly lower opioid consumption at 24 and 48 h, as well as a reduced number of postoperative compressions of the analgesic pumps and lower postoperative analgesic scores. These findings are consistent with the outcomes of the traditional combination block of RSB and TAPB [23–25]. There was no significant difference in the incidence of postoperative rescue analgesia among the three groups, which contrasts with the findings of the previous study [23]. There could be several reasons for this. First, some patients refused to use postoperative rescue analgesic drugs because of dizziness and other adverse reactions. Second, it might be that the patient’s stress response was effectively controlled due to the intraoperative administration of sufentanil and nerve blocks. Furthermore, the increased number of analgesic pump presses and sufentanil dosage may also affect the need for rescue analgesia. Third, it is possible that the sample size is too small, and the difference may not be statistically significant.

Postoperative pain, male gender, and catheter-related bladder discomfort were independent risk factors for EA [10, 27]. Wang et al. reported that infraorbital nerve block administered at the beginning of surgery significantly decreased the incidence of EA and duration of EA in children undergoing cleft lip repair surgery [28]. In this study, one puncture technique of RSB combined with TAPB also decreased the incidence of EA in the PACU, but the factors related reward further research. The most common method of managing postoperative pain is through the use of opioids, but this approach has side effects such as nausea, vomiting, urinary retention, and constipation [29]. Although there were no statistical differences between the three groups in terms of PONV, but in the aspect of dizziness, the times to first exhaust and first off-bed activity in the block group were shorter than those in the control group, indicating the advantages of regional nerve block and meeting the requirements of rapid rehabilitation.

The advantage of this study is that we have introduced a novel operation method, which can simultaneously perform RSB and TAPB blocks with one puncture. This simplifies the puncture process, reduces the number of puncture points, and offers a new multimodal analgesia option for upper abdominal surgery. Despite these advantages, this study also has several limitations. Firstly, the sample size in this study was small, and it was conducted at a single center. Secondly, the identification of insufficient intraoperative analgesia based on hemodynamics and BIS may not be enough, and we added sufentanil at the start of the operation and continued to administer it regularly during the procedure. This may explain the reason for no statistical difference in the amount of intraoperative sufentanil among the three groups in this study. Finally, our study shows that the combination of RSB and TAP block by one puncture is significantly more effective in providing analgesia compared to the subcostal TAP method. This finding is consistent with previous studies [9]. However, we did not compare the analgesic effect and block plane between the one-puncture method and the multipoint method, which will be the focus of our next research.

## Conclusions

In this study, we found that the combination of RSB and TAPB by one puncture technique in laparoscopic upper abdominal surgery significantly reduced postoperative pain and analgesic consumption, and facilitated postoperative recovery.

### Electronic supplementary material

Below is the link to the electronic supplementary material.

Supplementary Material 1

Supplementary Material 2

## Data Availability

The datasets used and/or analyzed during this study are available from the corresponding author upon reasonable request.

## Abbreviations

US: Ultrasound
TAPB: Transversus abdominis plane block
RSB: Rectus sheath block
EOM: External oblique muscle
IOM: Internal oblique muscle
TAM: Transverse abdominis muscle
LA: Local anesthetic
NS: Normal saline
PONV: Postoperative nausea and vomiting
ASA: American Society of Anesthesiologists
BMI: Body mass index
PCIA: Patient-controlled intravenous analgesia
VAS: Visual analogue scale
EA: Emergence agitation
RASS: Richmond Agitation Sedation Scale
BIS: Bispectral index
MAC: Minimal alveolar concentration
PACU: Post-anesthesia care unit
SD: Standard deviations
IQR: Interquartile range
