# Supplementary material for: Combined rectus sheath block with transverse abdominis plane block by one puncture for analgesia after laparoscopic upper abdominal surgery: a randomized controlled prospective study
Source: BMC Anesthesiol. 2024 Feb 9;24:58. doi: 10.1186/s12871-024-02444-6 (PMC10854179; doi:10.1186/s12871-024-02444-6)
Supplement: Supplementary file 1 — Supplementary Material 1 [file 12871_2024_2444_MOESM1_ESM.docx]

**Figure Legend**

**Additional file 1** MP4. Operating video under ultrasound of the one-puncture RSB+TAPB.

The probe was positioned below the costal margin and moved outward along the costal margin, revealing the RAM overlapping TAM. Under direct vision, we pierced the anterior layer of the posterior sheath and administered 15 mL of 0.33% ropivacaine. Then, the needle broke through the posterior layer of the tendon, 15 mL of 0.33% ropivacaine was slowly injected, and the needle tip was advanced within the expanded plane, which was enlarged by the local anesthetic to block a wider area.
